# Hemothorax caused by injury of musculophrenic artery after ultrasound-guided percutaneous liver biopsy: a case report

**DOI:** 10.1186/s13256-024-04619-8

**Published:** 2024-06-26

**Authors:** Jing-ru Yang, Sai Wu, Jian Li, Xiao-juan Tian, Zhuo-xi Xue, Xiao-yan Niu

**Affiliations:** 1 Department of Ultrasound, The Affiliated Hospital of Qingdao University, Qingdao, 266003 Shandong China; 2 Department of Interventional Radiology, The Affiliated Hospital of Qingdao University, Qingdao, 266003 Shandong China

**Keywords:** Hemothorax, Musculophrenic artery, Ultrasound, Percutaneous liver biopsy, Angiography

## Abstract

**Background:**

Hemorrhage is the most common major complication after liver biopsy. Hemothorax is one type of bleeding and is very rare and dangerous. Several cases of hemothorax subsequent to liver biopsy have been documented, primarily attributed to injury of the intercostal artery or inferior phrenic artery and a few resulting from lung tissue damage; however, no previous case report of hemothorax caused by injury of musculophrenic artery after liver biopsy has been reported.

**Case presentation:**

A 45-year-old native Chinese woman diagnosed with primary biliary cirrhosis due to long-term redness in urination and abnormal blood test indicators was admitted to our hospital for an ultrasound-guided liver biopsy to clarify pathological characteristics and disease staging. A total of 2 hours after surgery, the patient complained of discomfort in the right chest and abdomen. Ultrasound revealed an effusion in the right thorax and hemothorax was strongly suspected. The patient was immediately referred to the interventional department for digital subtraction angiography. Super-selective angiography of the right internal thoracic artery was performed which revealed significant contrast medium extravasation from the right musculophrenic artery, the terminal branch of the internal thoracic artery. Embolization was performed successfully. The vital signs of the patient were stabilized after the transarterial embolization and supportive treatment.

**Conclusion:**

This case draws attention to the musculophrenic artery as a potential source of hemorrhage after percutaneous liver biopsy.

## Background

Percutaneous ultrasound-guided liver biopsy is considered one of the most important diagnostic tools to evaluate diffuse liver diseases [1]. Hemorrhage is the most common major complication after liver biopsy, and bleeding of any kind has been reported to occur in up to 11% of patients [2]. Hemothorax is much less common than other types of hemorrhage and can be very dangerous and life-threatening [3]. The initial management of hemothorax should involve angiographic embolization, ensuring comprehensive screening of all potentially injured arteries including intercostal arteries and all possible blood supply arteries to the diaphragm [4]. Here, we report a case of hemothorax caused by injury of musculophrenic artery after percutaneous liver biopsy, which, to our knowledge, has never been reported.

## Case presentation

A 45-year-old native Chinese woman was diagnosed with primary biliary cirrhosis due to long-term redness in urination and abnormal blood test indicators. She had no diarrhea, vomiting, or abdominal pain. Vital signs and other physical examinations were normal. Liver function tests showed alanine transaminase (ALT) 104 U/L, aspartate transaminase (AST) 95 U/L, alkaline phosphatase (ALP) 269 U/L, gamma glutamyl transpeptidase (GGT) 440 U/L, and serum total bilirubin 23 µmol/L. Immunological tests were as follows: anti-mitochondria antibody (AMA) positive, AMA-M2-3E(BPO, a recombinant fusion protein that expresses three lipoyl-binding functional domains of BCOADH-E2,PDH-E2,OGDH-E2) positive(+++), and antinuclear autoantibodies (ANA) positive (++). The results of all hepatitis virus tests were negative. Ultrasound showed a coarse echotexture of the liver, and computed tomography (CT) showed abnormal liver morphology, with atrophy of the right lobe and enlargement of the left lobe. Both modalities displayed cirrhosis, but there were no focal hepatic lesions. She has had mitral valve replacement (bioprosthetic valve) for 1 year. She denied any other chronic illnesses, as well as neurological and psychiatric disorders. There is no known family history of hepatobiliary, gastrointestinal, or other genetic diseases. The patient was admitted to our hospital for an ultrasound-guided liver biopsy to clarify pathological characteristics and disease staging. The informed consent was obtained from the patient.

**Fig. 1:**
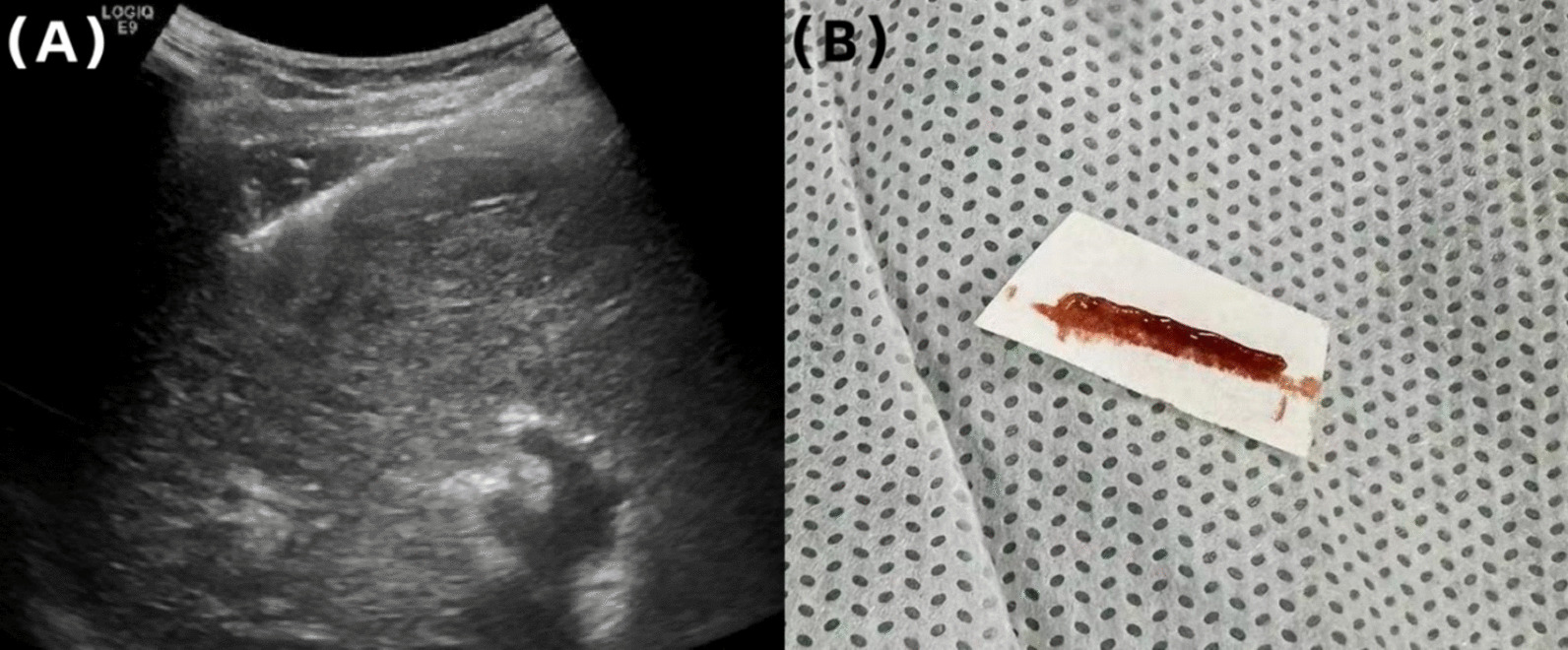
**A** Liver biopsy route. **B** Biopsy tissue of liver obtained

**Fig. 2:**
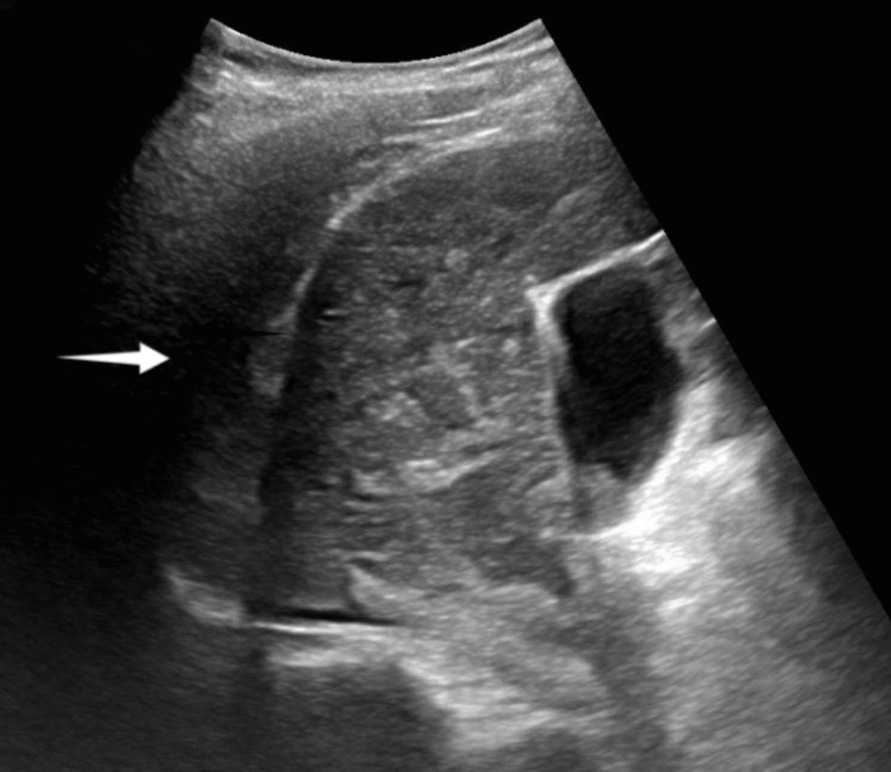
The US showed the right pleural effusion (white arrow), indicating the possibility of hemothorax

Preoperative tests showed that the hemoglobin, prothrombin time, bleeding time, and platelet count were normal and she was not taking any anticoagulants. Ultrasound-guided liver biopsy was carried out with GE Logiq E9 (GE Medical Systems Ultrasound and Primary Care Diagnostics, LLC, Wauwatosa, WI, USA) and a convex array probe (C6-1). The patient was in the left decubitus position and segment VI of the right lobe was chosen as the target of biopsy. A BARD automatic biopsy gun with an 18 G needle was applied and two strips of liver tissue were obtained (Fig. 1). During the puncture procedure, electrocardiogram monitoring was normal, blood pressure (BP) was 100/67 mmHg, heart rate (HR) was 80 beats per minute, and pulse blood oxygen saturation (SpO_2_) was 99% at the end of the surgery. Ultrasound (US) showed no active bleeding in the needle track. The patient was sent to the observation room for rest and postoperative monitoring, and the patient’s family members were asked to press the puncture site for at least 30 min. A total of 30 minutes after surgery, the patient began to complain of pain with breathing in the puncture area; the US showed no obvious fluid accumulation around the puncture point or in the pelvic/abdominal cavity. HR was 70 beats per minute, BP was 103/72 mmHg, SpO_2_ was 98%, and close observation was required. A total of 2 hours after surgery, the patient complained of discomfort in the right chest and abdomen, blood pressure dropped to 95/63 mmHg, HR was 68 beats per minute, and SpO_2_ was 97%. Ultrasound revealed an effusion in the right thorax, but preoperative CT showed no fluid accumulation, so we strongly suspected that the puncture caused hemothorax (Fig. 2). Urgent blood routine examination showed red blood cell (RBC) level of 3.77 × 10^12^/L and hemoglobin (Hb) of 120 g/L. Dextrose saline infusion was administered to help increase the effective blood volume, stabilize the blood pressure, and replenish energy. At the same time, hemocoagulase was given immediately to stop bleeding. Considering the possibility of arterial bleeding, the patient was referred to the interventional department for digital subtraction angiography (DSA).

**Fig. 3:**
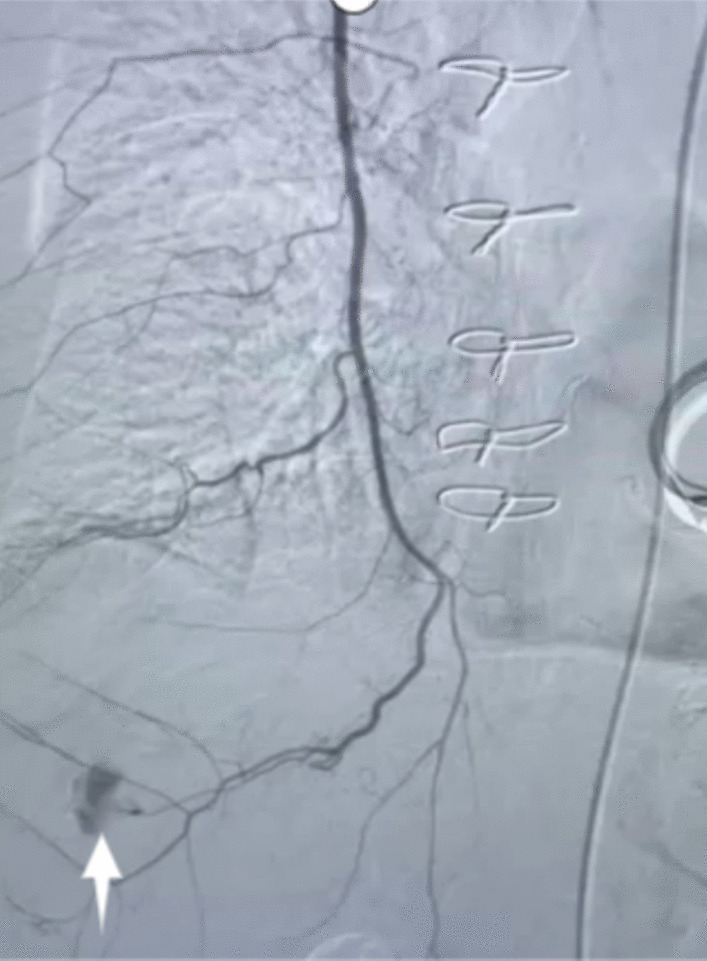
This showed contrast medium extravasation (white arrow) from the right musculophrenic artery, the terminal branch of the right internal thoracic artery

**Fig. 4:**
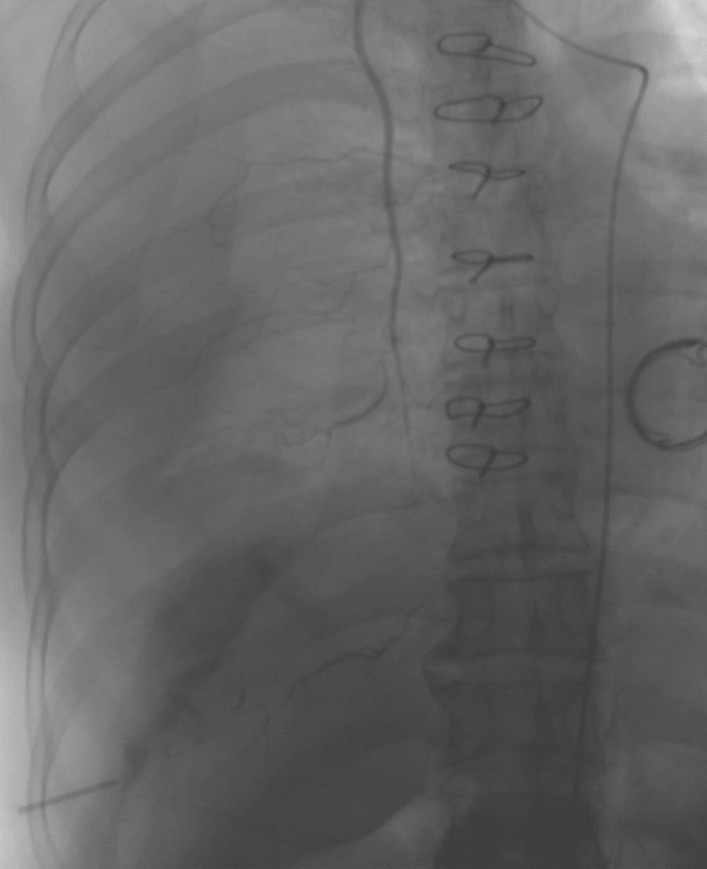
Embolization was performed successfully and the bleeding was stopped

Transcatheter angiography was performed immediately in the interventional operating room. Using a right femoral artery approach, a 4-F SIM1 catheter (Clinical Supply, Hashima, Japan) was selected to perform the angiography of the right 8–12th intercostal artery and the right inferior phrenic artery, and there was no leak of contrast medium. Angiography of liver arteries showed accumulation of a small amount of contrast medium and embolization was performed with 150–360 μm gelfoam. However, the source of the hemothorax has not been found, and the patient’s blood pressure continued to drop, and with continuous dopamine pumping, the blood pressure could be forced to maintain at 70/40 mmHg. The patient’s unstable vital signs and the delayed identification of the bleeding source had posed significant challenges and immense pressure on both sides of the medical team. Consequently, we had made a collective decision to explore alternative blood vessels that might potentially be responsible for the hemorrhage. Finally, super-selective angiography of the right internal thoracic artery was performed using a microcatheter, which revealed significant contrast medium extravasation from the right musculophrenic artery (Fig. 3). Embolization was performed with approximately 1.5 ml of iodized oil (Lipiodol) mixed with *N*-butyl-2-cyanoacrylate (NBCA) (Lipiodol: NBCA, 4:1) and duplicated angiography showed successful embolization (Fig. 4). The vital signs of the patient were stabilized after the transarterial embolization (TAE) and supportive treatment.

**Fig. 5:**
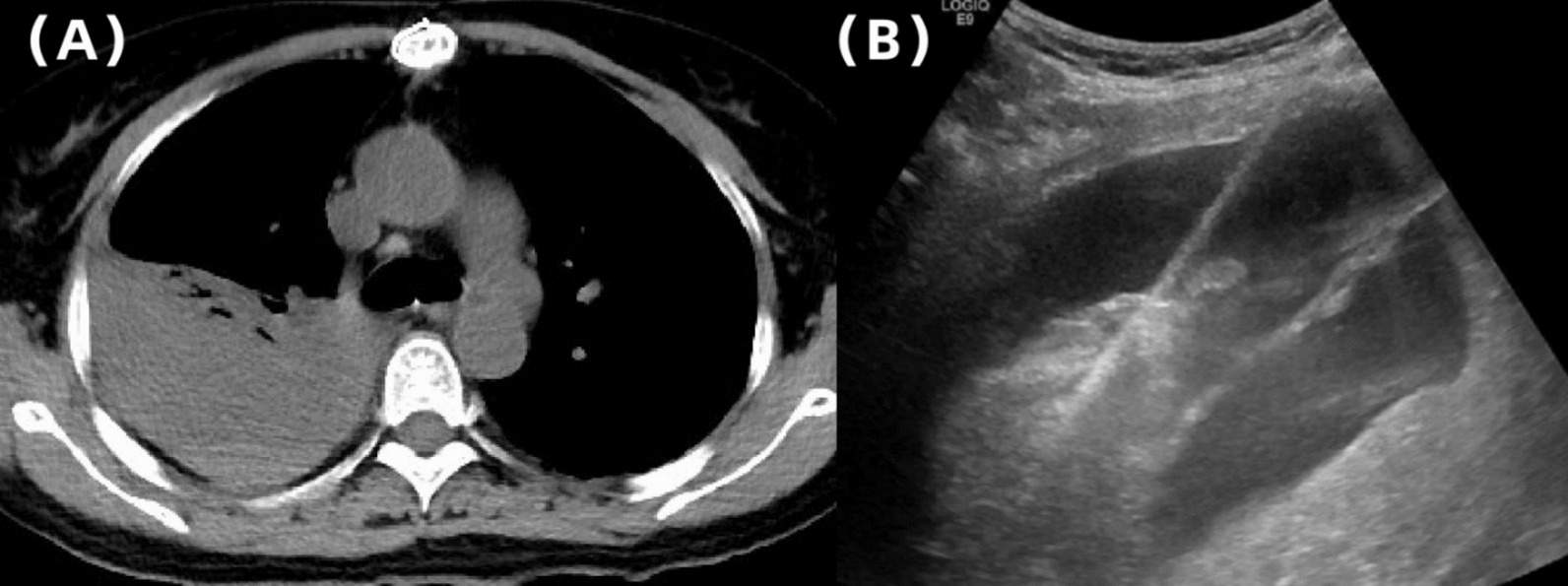
**A** Reexamination of chest computed tomography following transarterial embolization revealed a massive hemothorax on the right side. **B** A thoracic drain was inserted under ultrasound guidance

Reexamination of chest CT following TAE revealed a massive hemothorax on the right side, and a thoracic drain was inserted the next day (Fig. 5). A total of 2500 ml of bloody fluid was drained within 4 days. The biopsy pathology showed an advanced stage of primary biliary cirrhosis. The patient’s general condition stabilized, and then she was discharged on day 8 of hospitalization. The patient had a follow-up examination 3 months later, which showed no abnormalities in the abdominal and thoracic cavities, indicating satisfactory recovery.

## Discussion and conclusions

Liver biopsy is considered the most specific test for evaluating the etiology and severity of various hepatic disorders [2]. Percutaneous ultrasound-guided liver biopsy is currently the safest and most reliable method, and complications are infrequent, most of them being minor (pain and vasovagal reaction) [3]. Bleeding remains the most serious complication of liver biopsy, and massive bleeding can be life threatening [5, 6]. Post-biopsy hemorrhage requiring intervention occurs in 0.4% of adults on average [7–9].

Hemorrhagic complications of liver biopsy include intrahepatic or subcapsular hematoma, intraperitoneal bleeding, hemobilia, and hemathorax [3, 10]. Intrahepatic or subcapsular hematomas occur in 1–23% of cases and are usually small and asymptomatic, but larger hematomas may cause pain from stretching the liver capsule. Bleeding into the peritoneal cavity produces pain or hypotension [3, 9]. For these two types of bleeding, ultrasound can display a color blood flow signal on the needle track and show intrahepatic or subcapsular liquid echo, or intraperitoneal blood accumulation. Most bleeding can be controlled by hemostatic treatment, absolute bed rest, and close monitoring of vital signs. In this case, when the patient complained of pain at the puncture site, we initially suspected intrahepatic or subcapsular hematoma caused by puncture needle track bleeding. However, ultrasound showed no positive signs and no fluid in the abdominal cavity, so this type of bleeding was ruled out. But angiography of liver arteries showed accumulation of contrast medium in the liver later and embolization was performed, which reminds us that even in the absence of ultrasound findings indicating liver or intraperitoneal bleeding, we should not take it lightly and the patient should be closely observed to prevent the possibility of delayed bleeding.

Hemothorax has been noted as a rare complication of percutaneous liver biopsy, and now with the guidance of ultrasound, the risk of hemothorax has been further reduced greatly, but it remains highly perilous and life threatening once it occurs [2, 10, 11]. Patients who develop hemothorax usually undergo biopsy of the right hepatic lobe via an intercostal approach, especially patients with liver cirrhosis and right liver atrophy, because the puncture needle needs to pass through the intercostal space, costophrenic angle, and the diaphragm, and possibly damage the blood vessels along the puncture path [12]. A few such cases have been reported, most cases are usually caused by injury to the intercostal artery or phrenic artery, and few are caused by damage to the lung tissue [4, 9, 10, 13–16]. After the discovery of hemothorax in this case, we immediately considered the injury of the intercostal artery or the phrenic artery, but the angiography of the right 8–12th intercostal artery and the right inferior phrenic artery revealed no leak of contrast medium. However, at that time, the patient’s blood pressure continued to drop, which was difficult to maintain, indicating that the bleeding did not stop by itself, and the bleeding point should not be found. Then it occurred to us that there are three main groups of blood supplying arteries to the diaphragm, including the inferior phrenic artery (branch of the abdominal aorta), the musculophrenic artery (one of the terminal branches of the internal thoracic artery), and the superior phrenic artery (branch of the thoracic aorta), which form extensive collateral anastomosis in the diaphragm, of which the inferior phrenic artery is the main blood supplying vessel to the diaphragm. As reported in the current literature, the phrenic artery injury detected by angiography generally refers to the inferior phrenic artery. In some cases, it is impossible to distinguish which group of artery injured due to the use of thoracotomy for hemostasis [4, 10, 14, 16]. Is it possible that the other two groups of vessels were involved in this case? So the catheter was put into the subclavian artery and superselective angiography of the right internal thoracic artery was performed, which revealed bleeding from one of its terminal branches, the musculophrenic artery. No previous case report of hemothorax caused by injury of the musculophrenic artery after liver biopsy, to our knowledge, has been reported.

This case draws attention to the musculophrenic artery as a potential source of hemorrhage after percutaneous liver biopsy. Once suspected of hemothorax, the ultrasound physician should seek assistance from the interventional department as soon as possible to locate the bleeding point and promptly treat it with embolization. Except for the most commonly injured intercostal arteries, it is also necessary to identify all possible blood supply arteries to the diaphragm. While searching for a bleeding point, only by complete mapping of all major arterial branches of the abdominal aorta and the thoracic aorta can successful interventional radiology be achieved. Awareness of thoracic and abdominal vascular anatomy and analyzing all the possible blood vessels that may be damaged during the procedure is crucial for ultrasound and interventional physicians.

## Data Availability

Not applicable.

## Abbreviations

DSA: Digital subtraction angiography
TAE: Transarterial embolization
ALT: Alanine transaminase
AST: Aspartate transaminase
ALP: Alkaline phosphatase
GGT: Gamma glutamyl transpeptidase
AMA: Anti-mitochondria antibody
ANA: Antinuclear autoantibodies
CT: Computed tomography
Bp: Blood pressure
HR: Heart rate
SpO2: Pulse blood oxygen saturation
US: Ultrasound
RBC: Red blood cells
Hb: Hemoglobin
NBCA: *N*-Butyl-2-cyanoacrylate
